# Contribution of irisin pathway in protective effects of mandarin juice (*Citrus reticulata* Blanco) on metabolic syndrome in rats fed with high fat diet

**DOI:** 10.1002/ptr.7128

**Published:** 2021-05-04

**Authors:** Lara Testai, Marinella De Leo, Lorenzo Flori, Beatrice Polini, Alessandra Braca, Paola Nieri, Luisa Pistelli, Vincenzo Calderone

**Affiliations:** ^1^ Department of Pharmacy University of Pisa Pisa Italy; ^2^ Interdepartmental Research Center “Nutraceuticals and Food for Health” University of Pisa Pisa Italy; ^3^ Interdepartmental Research Centre of Ageing Biology and Pathology University of Pisa Pisa Italy

**Keywords:** *Citrus reticulata*, flavonoids, inflammation, irisin, metabolic syndrome, Rutaceae

## Abstract

The beneficial effects of *Citrus* fruits and their secondary metabolites on the cardiovascular system are well established. Moreover, growing evidence suggests beneficial role for prevention of obesity and related dysfunctions. *Citrus reticulata* Blanco (Rutaceae) is one of the most consumed *Citrus* fruits, but it is poorly investigated. Mandarin juice obtained from *C. reticulata* fruits, collected in the *Horti Simplicium* of Pisa Charterhouse, presents a high amount of flavanone glycosides, including hesperidin and a number of polymethoxyflavonoids, in particular nobiletin and tangeretin. On Wistar rats fed with a high fat diet for 21 days, mandarin juice significantly contained the percentage weight gain, reduced visceral adipose tissue and the inflammatory markers TNF and IL‐6. Furthermore, mandarin juice influenced irisin pathway, increasing its plasma levels. Finally, supplementation with mandarin juice contributed to improve mitochondrial membrane potential, partially compromised with high fat diet, making mitochondria less susceptible to harmful events, such as ischemia. Taken together, these results suggest that *C. reticulata*, through its main metabolites, is able to produce beneficial effects in metabolic syndrome and to promote browning process, through involvement of the novel interesting irisin pathway.

## 1 INTRODUCTION

Metabolic syndrome (MS) is a condition affecting at least 30% of adults in the Western society, but it is widespread also in the urban population of some developing countries (Saklayen, 2018). It is characterized by the presence of, at least, three following variables: elevated waist circumference, high levels of blood pressure, insulin resistance, and dyslipidemia (Sirtori, Pavanello, Calabresi, & Ruscica, 2017). MS is strongly associated with increased risk of developing diabetes and atherosclerotic and nonatherosclerotic cardiovascular diseases (Mottillo et al., 2010). Very recent studies highlighted a close correlation between MS and serum irisin levels: indeed, lower levels of this adipomyokine were found in the serum of patients with MS, as well as type 2 Diabetes Mellitus. Noteworthy, irisin is involved in white adipose tissue browning and in energy expenditure, insulin sensitivity, and antiinflammatory pathways, therefore it is emerging as a crucial player in metabolic disorders and as a possible target to mitigate these conditions (Huerta‐Delgado et al., 2020).

Beneficial effects of *Citrus* fruits on the cardiovascular system and the positive impact on blood pressure levels are well‐established (Testai & Calderone, 2017). Moreover, growing evidence suggests beneficial role of *Citrus* fruits and their secondary metabolites for prevention of obesity, improving type 2 diabetes condition, and in the reduction of dyslipidemia (De Leo et al., 2019; Lee et al., 2013; Morrow et al., 2020; Shen, Wan, Wang, & Jiang, 2019; Sundaram, Shanthi, & Sachdanandam, 2014). Antihyperlipidemic effects of bergamot (*Citrus bergamia* Risso & Poit.) have been well‐established and several formulations are commercially available to reduce hypercholesterolemia (De Leo et al., 2019; Landi et al., 2019; Maugeri, Ferlazzo, De Luca, Gitto, & Navarra, 2019).

As concern the active metabolites, the *Citrus* flavonoids, such as naringenin and nobiletin, have been associated with preventive effects against the development of metabolic disorders and atherosclerosis (Alam et al., 2014; Burke et al., 2018). Recently, another *Citrus* flavanone, hesperidin, has been identified as a regulator of both lipid and glucose metabolism through the activation of AMPK‐PPAR pathway (Xiong et al., 2019). These results lead to hypothesize that such beneficial effects can be attributed to active metabolites and therefore can be considered as property of other *Citrus* fruits. In particular, *Citrus reticulata* Blanco (Rutaceae) is one of the most widely consumed and distributed *Citrus* fruits all over the world (Wang et al., 2017). However, the knowledge of its cardiovascular profile is lacking. Previous investigation about the chemical content of different varieties of *C. reticulata* showed that, similarly to other *Citrus* fruits, flavedo, albedo, and juice are rich in flavonoids, such as flavones, flavanones, and polymethoxyflavones (Russo, Cacciola, Bonaccorsi, Dugo, & Mondello, 2011; Wang et al., 2017; Zhang et al., 2014), thus arousing a great interest in their potential biological value. The investigation on *C. reticulata* fruits, performed in this study, is combined to the valorization of an Italian cultural heritage, since the plant species was collected in the *Horti Simplicium* of Pisa Charterhouse, an ancient monastery located in Calci (Pisa, Italy). After the abandon of last monks at the beginning of the 1970s, it became a National Museum and hosted later the Natural History Museum of the University of Pisa. Therefore, in the context of the requalification of Pisa Charterhouse *Citrus* plants, the juice of *C. reticulata* has been evaluated for its in vivo protective effects on metabolic syndrome and its content in terms of bioactive substances.

## 2 MATERIALS AND METHODS

### 2.1 Chemicals

HPLC grade acetonitrile and formic acid were purchased from VWR (Italy). HPLC grade water (18 MΩ) was prepared by using a Mill‐Ω^50^ purification system (Millipore Corp., MA, USA). *N,N*‐dimethylformamide (DMF) was purchased from Alfa Aesar (Germany). Naringin and vitexin 2″‐*O*‐glucoside were isolated in our laboratory from plant materials during previous studies and fully characterized by 1D‐ and 2D‐NMR analyses.

### 2.2 Plant material

Fresh fruits of *Citrus reticulata* Blanco were collected in February 2017 in the *Horti Simplicium* of the monks of the Pisa Charterhouse (“Certosa di Pisa” located in Calci, Pisa, Italy) and identified by Prof. Paolo Emilio Tomei. A voucher specimen (N.A. 4,100 *Citrus reticulata*/033389) was deposited at Herbarium Horti Botanici Pisani (Pisa, Italy).

The mandarin juice (MJ) was obtained by a manual juicer and then stored at −20°C for both chemical analyses and biological test.

### 2.3 Chemical characterization of mandarin juice by HPLC‐PDA/UV‐ESI‐MS/MS

Defrosted juice (10 mL) was mixed to an equal volume of DMF, centrifuged three times for 5 min at 1,145×*g*, then the supernatant was filtered through PTFE membrane (3 mm, 0.45 μm) before HPLC analyses. The LC‐photodiode array (PDA)‐mass spectrometry (MS) system was composed by a Surveyor autosampler and a Surveyor LC pump, interfaced with a Surveyor PDA/UVvis detector and a LCQ Advantage ion trap mass spectrometer equipped with an electrospray ionization (ESI) source (ThermoFinnigan, CA, USA). A Synergi Fusion‐RP column (4.6 × 250 mm, 4 μm, Phenomenex, Italy) was used for chromatography, eluting with a mixture of acetonitrile (solvent A) and water (solvent B) using the solvent gradient reported by Da Pozzo et al. (2018). Elution (20 μL injection volume) was performed at 0.8 mL/min flow rate, with a splitting system of 2:8 to MS detector (160 μL/min) and PDA detector (640 μL/min), respectively. PDA/UV spectra were acquired in a wavelength range of 200–600 nm, with 254, 280, and 325 nm as preferential channels. Mass spectra were registered in both ESI negative and positive ion modes in a scan range of *m/z* 150–2000. Negative ionization parameters were optimized as previously reported (Da Pozzo et al., 2018), while in the positive ion mode the parameters were as follows: source voltage 4.50 kV, capillary voltage 29.00 V, tube lens offset 50.00 V, capillary temperature 250°C, sheath gas (N_2_) flow 60.00 arbitrary units, auxiliary gas (N_2_) flow 3.00 arbitrary units. The amount of flavonoids was determined constructing two calibration curves by using naringin as external standard for flavonoid *O*‐glycosides and polymethoxyflavones, and vitexin 2”‐*O*‐glucoside as external standard for flavonoid *C*‐glycosides. Stock solutions (1.0 mg/mL in DMF) were prepared for each standard and then five different concentration levels were obtained by serial dilution in a range 0.008–0.5 mg/mL. Each solution and mandarin juice were analyzed by triplicate injections. Calibrations curves were obtained plotting integrated UV peak areas at 325 and 280 nm for naringin and vitexin 2″‐*O*‐glucoside, respectively, against the standard solutions concentrations (mg/mL). Relation between variables was established by linear simple correlation. For standard linear regression, *R* ^2^ was 0.9994 for naringin and 0.9998 for vitexin 2″‐*O*‐glucoside. The flavonoid amount of mandarin juice was finally expressed as mg/L of fresh juice. Chromatografic, UV and MS data were elaborated using Xcalibur 3.1 software, while quantitative analyses were obtained using a GraphPad Software Prism 6.0.

### 2.4 Animals

In vivo experiments were carried out according to European (EEC Directive 2010/63) and Italian (D.L. March 4, 2014 n.26) legislation (491/2018‐PR); moreover, ARRIVE guidelines have been put into practice. Animals were housed in cages with food and water ad libitum, and they were exposed to a 12 h dark/light cycle. The experiments were conducted on male Wistar rats (ENVIGO) with a body weight of 336 ± 8 g.

### 2.5 Chronic in vivo treatment

The animals were randomly divided into three groups (6 animals per group) and treated for 21 days: one group was treated with a standard diet (STD, ENVIGO; for composition Table 1). The second one was treated with a high fat diet (HFD, SAFE; for composition see Table 1). The third one was treated with HFD + mandarin juice 24% v/v (HFD + MJ) diluted in water. The dosage of mandarin juice has been established on the basis of previous epidemiological and clinical studies in which other *Citrus* juices have been used (Morand et al., 2011; Testai & Calderone, 2017). The water intake was evaluated daily, instead the body weight and the food intake three times a week for each animal.

**TABLE 1:** Composition of the two diets used (STD and HF)

	STD	HF
Protein (%)	14.3	12.9
Fat (%)	4.0	19.2
Carbohydrate (%)	48.0	50.2
Calories from protein (%)	20.0	12.1
Calories from fat (%)	13.0	40.7
Calories from carbohydrate (%)	67.0	47.2
Na (mg/kg)	1,000.0	2,234.1
K (mg/kg)	6,000.0	5,284.3
Mg (mg/kg)	2,000.0	1,294.0
Ca (mg/kg)	7,000.0	6,312.5
Mn (mg/kg)	100.0	47.7
Fe (mg/kg)	175.0	253.2
Cu (mg/kg)	15.0	18.5
Zn (mg/kg)	70.0	48.7
P (mg/kg)	6,000.0	5,144.2
Cl (mg/kg)	3,000.0	3,508.1
Vitamin A (IU/g)	6.0	7.4
Vitamin E (IU/kg)	120.0	95
Vitamin D_3_ (IU/g)	0.6	1.0
Vitamin K_3_ (mg/kg)	20.0	13.3
Vitamin B_1_ (mg/kg)	12.0	4.2
Cholesterol (mg/kg)	—	12,488

At the end of the treatment each animal was deprived of food and after 24 h was anesthetized with Thiopental Sodium (70 mg/kg, MSD animal health). The blood was collected, through the caudal vein, to perform blood glucose test (Glucocard G meter, Menarini Diagnostics). Subsequently the animals were sacrificed with an overdose of Thiopental Sodium. Blood was collected in tubes with the anticoagulant EDTA (BD Vacutaine) by intracardiac sampling and organs (heart, liver, and abdominal adipose tissue) were removed, weighed, and stored for functional and enzymatic investigations.

### 2.6 Evaluation of blood lipid panel

Blood was immediately used for the evaluation of lipid panel (total cholesterol, HDL, LDL, triglycerides) and glycated hemoglobin levels using Cobas b101 instrument (Roche Diagnostics, Milan, Italy). Residue samples were centrifuged at 1,000×*g* for 10 min, obtaining plasma, which was frozen at −20°C for subsequent analysis.

*Data analysis*. Blood samples were collected from six animals per group and results were expressed as mean ± SEM. Cardiovascular risk was calculated as ratio between total cholesterol and HDL cholesterol levels. One‐way ANOVA followed by Bonferroni's post hoc test was used to compare groups for statistical differences (*p* < .05).

### 2.7 Functional analysis of cardiac mitochondrial membrane potential

#### 2.7.1 Cardiac mitochondria isolation

The heart was cut into small 2/3 mm^3^ pieces in STE (composition: sucrose 250 mM, Tris 5 mM, EGTA 1 mM; pH 7.4) and constantly on ice (4°C). The pieces were transferred in STE (4°C) and then homogenized with Ultra‐Turrax (model: IKA, T‐18 Basic). After three homogenization cycles of about 20 s, the first centrifugation was carried out: 1,090×*g*, 3 min, 4°C. The supernatant was transferred to a new tube kept on ice and the pellet was resuspended in STE. The suspension obtained was centrifuged again under the same conditions. Then, the supernatants were picked up and centrifuged again but under different conditions: 11,970×*g*, 10 min, 4°C. Two pellets were obtained and the supernatants were discarded. Each of them was resuspended in STE and centrifuged again at 11,970×*g* for 10 min, 4°C. The pellet was preserved and resuspended in ST (composition: sucrose 250 mM, Tris 5 mM; pH 7.4) and centrifuged using the previous conditions. The pellet obtained, corresponding to the mitochondrial fraction, was resuspended in 400 μL of ST and transferred to a frozen eppendorf. The mitochondrial protein concentration in the supernatant was determined spectrophotometrically by Bradford assay (Bio‐Rad, USA), using a microplate reader (EnSpire, PerkinElmer, USA).

#### 2.7.2 Measurement of mitochondrial membrane potential (ΔΨm)

The membrane potential (∆Ψm) of the isolated mitochondria was determined using a potentiometric method. The lipophilic cation tetraphenylphosphonium (TPP^+^), used for this procedure, was detected with a TPP^+^ sensitive mini electrode (WPI, TipTPP, USA), coupled to a reference electrode (WPI, FL, US) and using a data acquisition software (Biopac Inc California, USA). Then, mitochondria (1 mg protein/mL) were added to the incubation buffer (composition: KCl 120 mM, K_2_HPO_4_ 5 mM, Hepes 10 mM, succinic acid 10 mM, MgCl_2_ 2 mM, TPP^+^Cl^−^ 10 μM; pH 7.4), and were continuously stirred with a small magnet to generate a mitochondrial suspension.

*Data analysis*. The membrane potential value was calculated according to the following experimental equation derived from Nernst equation:

$$∆\varphi_{m}=60\;x\;\log\frac{\frac{V_{0}{\left[{\mathrm{TPP}}^{+}\right]}_{0}}{{\left[{\mathrm{TPP}}^{+}\right]}_{t}}-V_{t}-K_{0}P}{V_{m}P+K_{i}P}$$

where ∆Ψm is the mitochondrial membrane potential (mV), *V*_0_ is the volume of the incubation medium before the addition of mitochondria, *V*_t_ is the volume of the incubation medium after the addition of the mitochondria, *V*_m_ is the volume of the mitochondrial matrix (taken as 1 μL/mg protein), [TPP^+^]_0_ and [TPP^+^]_t_ represent, respectively, the TPP^+^ concentrations recorded before addition and at time *t*, *P* is the protein concentration expressed in mg, *K*_0_ and *K*_i_ are apparent external and internal partition coefficients of TPP^+^ (14.3 and 7.9 μL/mg, respectively). The mitochondrial membrane potential was evaluated on six different animals per group, and data were expressed as mean ± SEM. One‐way ANOVA followed by Bonferroni's post hoc test was used to compare groups for statistical differences (*p* < .05).

### 2.8 Evaluation of citrate synthase activity on adipose tissue

Frozen adipose tissues were homogenized on ice with an ultra‐turrax homogenizer (IKA‐Werke GmbH & Co., Germany) in a cold buffer (composition: sucrose 250 mM, Tris 5 mM, EGTA 1 mM, Triton X‐100 0.02%; pH 7.4). Then, homogenates were centrifuged at 12,000×*g* for 15 min at 4°C (EuroClone, Speed Master 14 R centrifuge, Italy). The supernatant was used for the citrate synthase (CS) activity determination, and the protein concentration in the supernatant was determined spectrophotometrically by Bradford assay (Bio‐Rad, USA), using a microplate reader (EnSpire, PerkinElmer, USA). Then, proteins were diluted in Tris‐buffer (Trizma base 100 mM, pH 8.2) containing 5,5′‐dithiobis‐(2‐nitrobenzoic) acid (DTNB, 100 μM) and acetyl‐coenzyme A (100 μM). The assay was performed in 96 multi‐well plates (1 μg of proteins per well) and the reaction was initiated by addition of oxaloacetic acid 500 μM. The absorption of the reaction product was measured spectrophotometrically at 30°C and 412 nm every 30 s for 15 min. Citrate synthase activity was determined by comparing the activity in the samples to that of known concentrations of the isolated enzyme (Sigma‐Aldrich, USA).

*Data analysis*. The assay was performed on six animals per group. Citrate synthase activity was expressed in mU/μg protein. Data were analyzed by a computer fitting procedure (software: GraphPad Prism 5.0). Results were expressed as mean ± SEM. One‐way ANOVA followed by Bonferroni's post hoc test was used to compare groups for statistical differences (*p* < .05).

### 2.9 Evaluation of inflammatory markers

The levels of TNF and IL‐6 were evaluated in adipose tissues using ELISA commercial kits (AbFRONTIER, Korea). About 500 mg of adipose tissue have been thawed, added with 500 μL of STE (pH 7.4, 4°C), and homogenized with potter. The suspension was transferred to an eppendorf and centrifuged for 15 min, at 12,000×*g* and 4°C. The supernatant was used to make the appropriate dilutions.

*Data analysis*. One‐way ANOVA followed by Bonferroni's post hoc test was selected as statistical analysis, and the difference between groups was considered statistically different when *p* < .05.

### 2.10 Investigation of irisin pathway

Irisin's levels were measured directly in plasma using an ELISA commercial kit (Adipogen Life Science, Switzerland). For subsequent passages of the ELISA kits, the protocols indicated by the manufacturer were followed.

Moreover, the downstream mediators were evaluated in adipose tissue (about 100 mg) by using the RNeasy Mini Kit (Qiagen, Germany), according to the manufacturer's protocol.

Then, total RNA was extracted and converted into first‐strand cDNA by using the QuantiNova Reverse Transcription Kit (Qiagen, Germany). QuantiNova SYBR Green kit (Qiagen, Germany) and specific primers for each molecule under evaluation (QuantiNova LNA PCR Assays, Qiagen, Germany) were used for qPCR experiments performed in a CFX Connect Real‐Time PCR System (Bio‐Rad Laboratories, Italy). In detail, the following primers were selected from GeneGlobe database: #2373737 with GeneGlobe Id – SBR1129530 for UCP‐1 gene, #2375731 with GeneGlobe Id – SBR1131524 for PPARG, #2436168 with GeneGlobe Id – SBR1191958 for PPARGC1A, and #2411078 with GeneGlobe Id – SBR1166871 for ACTB. The transcriptional expression levels were calculated by the comparative critical threshold (C_T_) method. To avoid possible variations related to cDNA input or presence of PCR inhibitors, the endogenous housekeeping β‐actin was quantified for each sample, and data normalized accordingly.

*Data analysis*. One‐way ANOVA followed by Bonferroni's post hoc test was selected as statistical analysis, and the difference between groups was considered statistically different when *p* < .05.

## 3 RESULTS AND DISCUSSION

### 3.1 Effect of mandarin juice on HF‐induced metabolic syndrome

A diet containing high levels of saturated fats contributes to significantly increase the body weight. Such type of diet represents a well‐known experimental model to reproduce metabolic syndrome in rodents. Indeed, several animal models confirm the relationship among high fat diet, body fat accumulation, and metabolic complications (Moreno‐Fernández et al., 2018).

**FIGURE 1:**
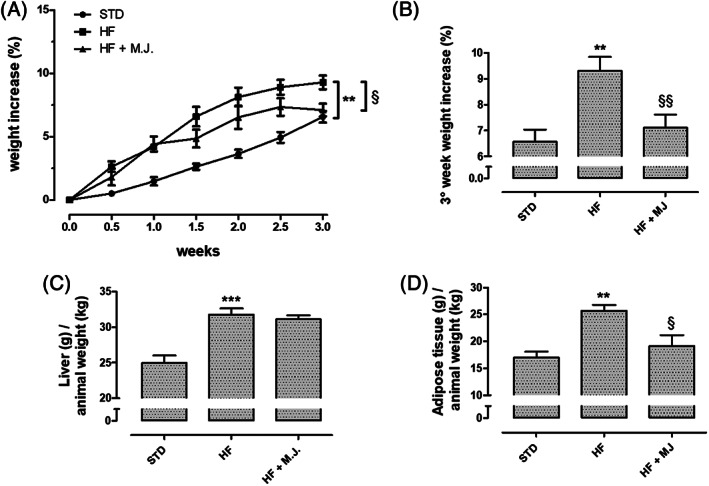
Effects of mandarin juice on body weight and tissue mass. (a) Body weight gain (%) during 3 weeks‐treatment. (b) Body weight gain (%) at the third week of treatment. (c) Liver weight (g) in relation to the body weight (kg) at the end of the 3 weeks of treatment. (d) Weight of the visceral white adipose tissue (g) in relation to the body weight (kg) at the end of 3 weeks of treatment. * indicates a significant statistical difference between HF and STD groups. **§** indicates significant statistical difference between HF and HF + MJ. The single symbol corresponds to *p* < .05; the double symbol to *p* < .01, and the triple symbol to *p* < .001

Accordingly, animals fed with STD diet for 3 weeks showed an increase of the body weight of 6 ± 0.5% (final weight of 358 ± 9 g); conversely, at the end of the treatment (21th day), the animals fed with HFD showed a body weight gain of about 9 ± 0.5% (final weight of 367 ± 8 g). The supplementation with MJ contained the weight gain if compared with HFD; in particular, at the 21th day the values were superimposable STD group (final weight of 360 ± 9 g; Figure 1a,b). In this study, the typical accumulation of fat in liver and visceral adipose tissue was observed in HFD group. Supplementation with MJ did not ameliorate fatty liver (Figure 1c) but significantly prevented the increase of visceral adipose tissue (Figure 1d).

According with the literature, rats fed for 21 days with HFD showed significantly higher levels of blood glucose, total cholesterol, triglycerides, and LDL if compared with STD diet (Table 2). Moreover, HDL value was markedly reduced (Sullivan, Cerda, Robbins, Burgin, & Beatty, 1993). MJ did not improve HDL, but clearly reduced blood glucose, total cholesterol, triglycerides, and LDL levels, significantly containing cardiovascular risk (Table 2). This improvement of lipid profile has been also described for other *Citrus* fruits, among which bergamot (De Leo et al., 2019; Lamiquiz‐Moneo et al., 2019). The positive effect on both body weight and visceral fat accumulation has been also described for other *Citrus* fruits, especially for those containing the amine synephrine (Barreca et al., 2017). However, synephrine has been found in the peel of bitter orange (*Citrus* x *aurantium* L.) and other *Citrus* fruits but, to our knowledge, it is not present in *C. reticulata* and especially in juice. An anorexigen effect of MJ can be excluded, since the daily intake was similar for all the treatment groups (data not shown). Chou and colleagues observed that a supplementation with an extract obtained from immature *C. reticulata* peel promoted antiobese effects and improved cardiometabolic profile in similar experimental conditions (Chou, Ho, & Pan, & M. H., 2018).

**TABLE 2:** Lipid and glycemic profile about STD, HF, HF + MJ groups

	STD	HF	HF + MJ
Total cholesterol (mg/dL)	80.0 ± 5.2	113.8 ± 5.3***	87.4 ± 3.6^**§§**^
Triglycerides (mg/dL)	69.0 ± 4.1	110.2 ± 15.6*	66.0 ± 3.0^**§§**^
Cholesterol HDL (mg/dL)	52.8 ± 5.1	25.6 ± 2.5***	26.0 ± 2.1
Cholesterol LDL (mg/dL)	6.8 ± 2.2	63.0 ± 2.8***	48.4 ± 3.6^**§**^
Cholesterol non‐LDL (mg/dL)	27.0 ± 2.1	88.0 ± 3.5***	61.4 ± 3.6^**§§§**^
Cardiovascular risk (Total Chol./Chol. HDL)	1.6 ± 0.1	4.5 ± 0.3***	3.4 ± 0.3^**§**^
Blood glucose (mg/dL)	64.5 ± 7.3	102.5 ± 5.2**	72.8 ± 5.4^**§**^

*Note*: * indicates a significant statistical difference between HF and STD groups. **§** indicates significant statistical difference between HF and HF + MJ The single symbol corresponds to *p* < .05; the double symbol to *p* < .01, and the triple symbol to *p* < .001.

**FIGURE 2:**
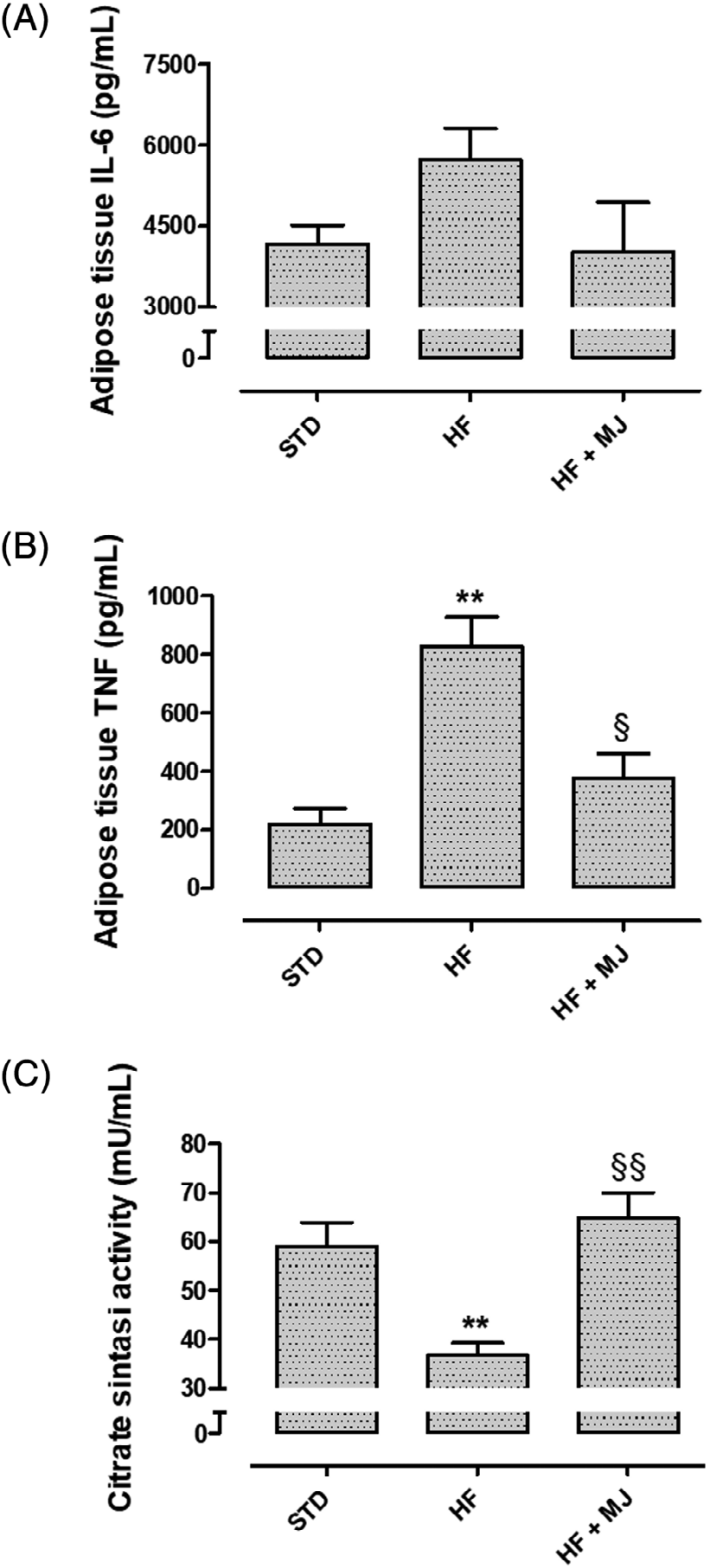
Effects of mandarin juice on pro‐inflammatory cytokines and cell metabolism. (a) Change of IL‐6 levels in adipose tissue. (b) Change of TNF levels in adipose tissue. (c) Activity of the Citrate Synthase enzyme in adipose tissue. * indicates a significant statistical difference between HF and STD groups. **§** indicates significant statistical difference between HF and HF + MJ. The single symbol corresponds to *p* < .05; the double symbol to *p* < .01, and the triple symbol to *p* < .001

A well‐described condition associated with metabolic disorders, such as obesity, is represented by the secretion of adipokines, chemokines, and cytokines, including TNF and IL‐6, from adipose tissue. This condition is associated to systemic inflammation, defined meta‐inflammation, implicated in metabolic and cardiovascular dysfunctions (Tan & Norhaizan, 2019). CS is a mitochondrial enzyme deeply involved in cell metabolism, being enrolled in the key step of Krebs cycle and it is considered a reliable indicator of energetic state. Interestingly, in this experimental model, in adipose tissue of rats fed with HFD, higher levels of pro‐inflammatory cytokines, IL‐6 and TNF were observed (Figure 2a,b). Conversely, CS activity was compromised. Oral supplementation with MJ clearly contributed to reduce cytokine release and improve the CS activity, suggesting that such a nutraceutical intervention could ameliorate metabolism and contain inflammation (Figure 2c).

**FIGURE 3:**
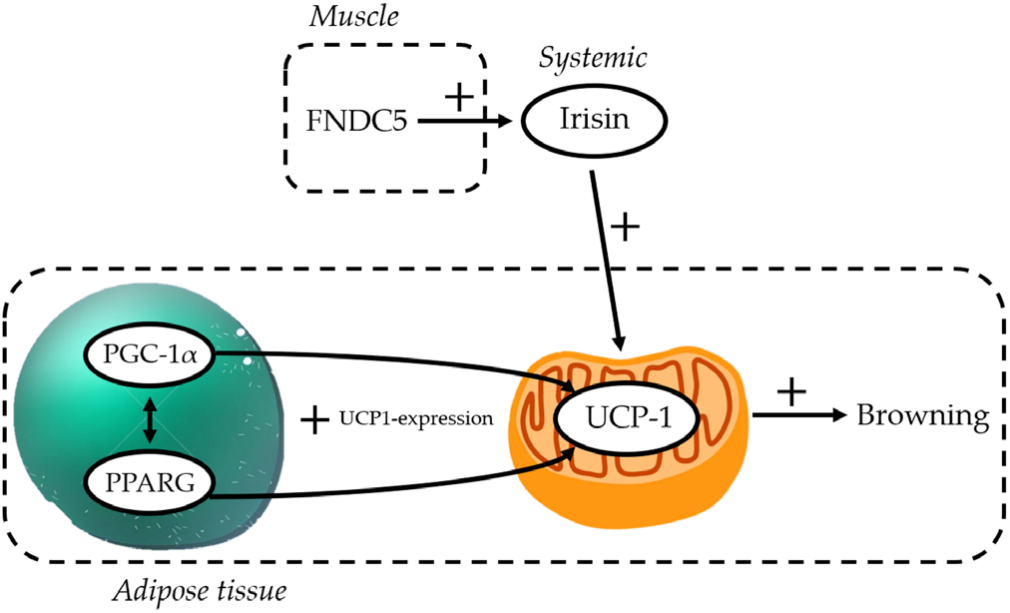
Schematic representation of the irisin pathway involved in the browning of white adipose tissue [Colour figure can be viewed at wileyonlinelibrary.com]

Growing evidence points out the key role of irisin in several metabolic disorders, including obesity (Flori, Testai, & Calderone, 2021). Irisin is a newly characterized myokine that promotes energy expenditure by white adipose tissue browning; indeed, irisin, secreted from skeletal muscle to blood, is responsible, through the stimulation of UCP‐1, for thermogenesis, a well‐known mechanism to loss body weight. Therefore, this discovery has generated great interest, being a possible exciting tool to act on the production of diet‐associated pro‐inflammatory cytokines, among which IL‐6 (Arhire, Mihalache, & Covasa, 2019). Indeed, HF diet is associated to reduced circulating irisin levels and to a downregulation of FNDC5 gene expression (whose proteolytic cleavage releases irisin) (Moreira de Macêdo et al., 2017). Besides FNDC5 and UCP‐1, several papers report a positive association between irisin secretion and PPAR‐γ coactivator 1‐alpha (PGC‐1α) stimulation (Flori et al., 2021). Indeed, PGC‐1α is a master regulator of mitochondrial biogenesis, that contributes to increase mitochondrial ATP synthesis and oxidative metabolism, such as oxidative phosphorylation and fatty acid β‐oxidation; its levels are reduced in HF diet if compared to low fat diet (Kazeminasab et al., 2018). Finally, peroxisome proliferator‐activated receptor γ (PPARγ) has been also found to promote mitochondrial biogenesis in adipocytes, accompanied with increased thermogenesis and browning (Liu, Wang, & Lin, 2019). Finally, it is noteworthy that PPARγ can also induce UCP‐1 expression. Taken together, an intricate network regulating the fat metabolism and promoting browning of white adipose tissue are emerging (De Oliveira Bristot, de Bem Alves, Cardoso, da Luz Scheffer, & Aguiar, 2019; schematic representation is reported in Figure 3).

**FIGURE 4:**
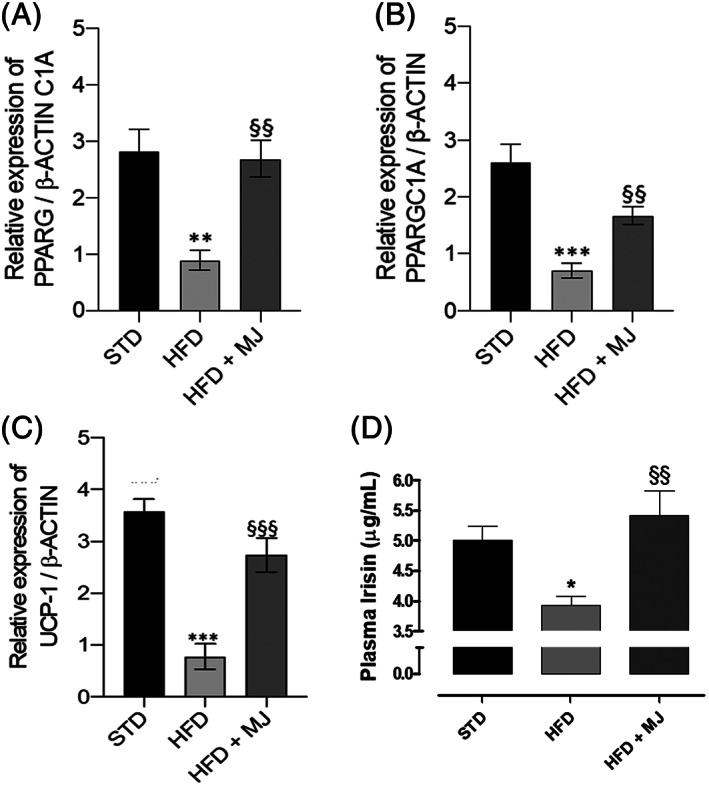
Mandarin juice‐induced changes in irisin pathway. (a) Change of transcriptional levels of PPARG, gene coding PPARγ, in adipose tissue; (b) Change of transcriptional levels of PPARGC1A, gene coding PGC‐1α, in adipose tissue; (c) Change of transcriptional levels of UCP‐1 in adipose tissue; (d) Change in serum irisin levels. * indicates a significant statistical difference compared to HF group. The single symbol corresponds to *p* < .05, the double symbol to *p* < .01, and the triple symbol to *p* < .001. * indicates a significant statistical difference between HF and STD groups. **§** indicates significant statistical difference between HF and HF + MJ. The single symbol corresponds to *p* < .05; the double symbol to *p* < .01, and the triple symbol to *p* < .001

Accordingly, lower levels of irisin have been measured in the blood of HFD‐treated animals. In contrast, irisin levels in HFD + MJ group were almost equivalent to that recorded in STD group (Figure 3d). Moreover, the supplementation with MJ counteracted in a significant manner the HFD‐induced decrease in the transcriptional expression levels of PPARG and PPARGC1A genes (Figure 4a,b), coding, respectively for PPARγ and PGC‐1α.

In addition to the decreased expression of PPARγ and PGC‐1α and the circulating irisin levels, HF diet induced also a significant decrease of transcriptional levels of UCP‐1 (Figure 4c). This decrease was almost absent in the adipose tissue from MJ‐treated rats, confirming the role of irisin pathway as a target in the effects of MJ, perfectly in agreement with the increase in UCP‐1 gene expression and UCP‐1 protein abundance observed in the classical brown adipose tissue depots (Zhang et al., 2016).

Therefore, such results suggest that the nutraceutical supplementation with mandarin juice can stimulate the browning mechanism on adipose tissue through the stimulation of irisin pathway.

**FIGURE 5:**
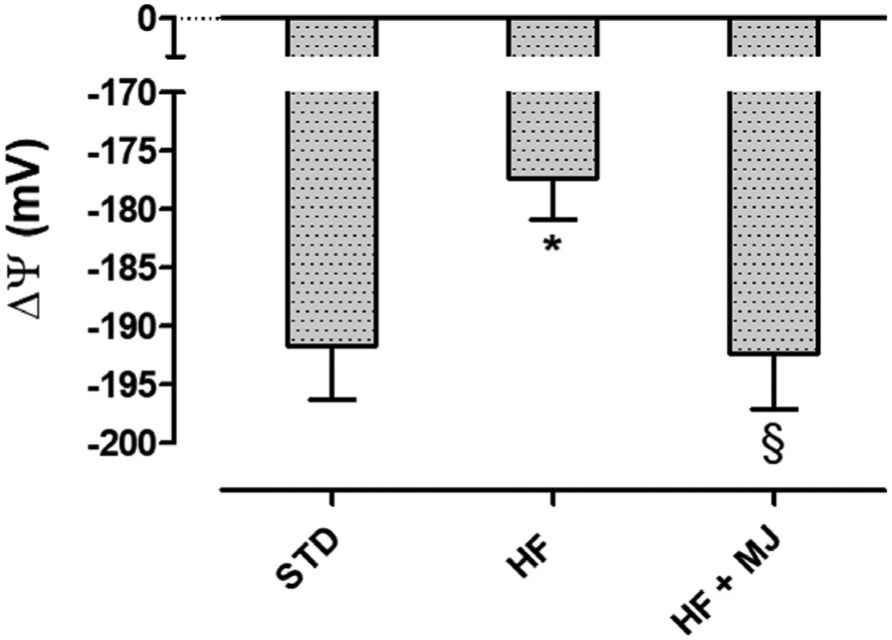
Effects of mandarin juice on cardiac mitochondrial membrane potential. * indicates a significant statistical difference between HF and STD groups. **§** indicates significant statistical difference between HF and HF + MJ. The single symbol corresponds to *p* < .05; the double symbol to *p* < .01, and the triple symbol to *p* < .001

Considering that cardiovascular diseases are the main consequences of metabolic syndrome and obesity (Galassi, Reynolds, & He, 2006), the cardiac mitochondrial membrane potential was evaluated as an indicator of cardiomyocytes tolerance toward possible harmful events. Indeed, modest, but significant, depolarization of mitochondrial membrane has been recognized in hearts of HFD‐fed animals and it has been associated with a lower bioenergetic efficacy. This makes mitochondria more susceptible to ischemic events. Interestingly, cardiac mitochondria from rats fed with HFD + MJ showed an inner membrane potential almost equal to organelles of STD group (Figure 5).

### 3.2 Chemical profile of mandarin juice

The chemical investigation on mandarin juice was carried out by means of HPLC‐PDA/UVvis‐MS/MS techniques. In total, 14 flavonoids were tentatively identified comparing their elution order, UV and ESI‐MS data (Table 3) with those reported in previous studies (Russo et al., 2011; Wang et al., 2017; Zhang et al., 2014). In particular, the fragmentation pathways of detected compounds generated in MS/MS experiments were useful to establish the structures of phenolics, in agreement with their presence in *Citrus* fruits. The identified flavonoids occurred in mandarin juice both as glycosides (**1**–**7**) and aglycones (**8**–**14**). Among flavonoid glycosides, flavone *C*‐glucosides apigenin (**1**) and diosmetin (**2**, **3**, and **5**) derivatives, flavanone *O*‐glycosides naringenin (**4**) and isosakuranetin (**8**) derivatives, flavone *O*‐glycosides hesperetin (**6**) and diosmetin (**7**) derivatives, were found. Some compounds were not unambiguously identified because they occur in *Citrus* fruits as isomers. Indeed, naringin and narirutin (compound **4**, M = 580 u), neohesperidin and hesperidin (compound **6**, M = 610 u), neodiosmin and diosmin (compound **7**, M = 608 u), and poncirin and neoponcirin (compound **8**, M = 592 u), differ from each other in the disaccharide chain, composed by the two isomers neohesperidose and rutinose, respectively. In all cases, the couples of flavonoid isomers showed the same fragmentation patterns in both negative and positive ion modes (Table 3), thus they were not distinguishable on the basis of MS data. Moreover, Wang et al. (2017) reported the occurrence of all compounds in the juice sacs of different Chinese mandarin varieties, while in Italian mandarin juices analyzed by Russo et al. (2011) narirutin, neoponcirin, and hesperidin were reported. The HPLC‐DAD analyses of Zhang et al. (2014) led to the identification in the juice sacs of a *C. reticulata* cultivar of naringin, hesperidin, and neohesperidin, with the last one the most abundant. Finally, diosmin and/or neodiosmin (compound **7**) were not reported in the cited literature. These differences can be explained by different varieties, origins, growing conditions, and other environmental factors related to the studied fruits (Luo et al., 2019). The flavonoid aglycones found in the juice herein analyzed were detected by ESI‐MS experiments in positive ion mode and all were found to be polymethoxyflavonoids (**9**–**14**), with nobiletin (**12**) and tangeretin (**14**) the most representative. Previous studies (Russo et al., 2011; Wang et al., 2017) did not report the presence of these compounds in the juice, but only in the *C. reticulata* flavedo (Wang et al., 2017; Zhang et al., 2014), while they were found in clementine cultivars (Sentandreu, Izquierdo, & Sendra, 2007).

**TABLE 3:** Chromatographic (retention time, *t*_R_), UV and ESI‐MS/MS data of flavonoids detected in *Citrus reticulata* juice

N	Compound	*t*_R_ (min)	*M*	+ESI‐MS/MS (*m/z*)^a^	−ESI‐MS/MS (*m/z*)^b^	λ_max_ (nm)
	*Flavone C‐glucosides*					
**1**	Vicenin‐2 (apigenin 6,8‐di‐*C*‐glucoside)	10.7	594	575, 503, **473**, 383, 353	**577**, 559, 541, 529, 511, 457	272, 335
**2**	Lucenin‐2 4′‐methyl ether (diosmetin 6,8‐di‐*C*‐glucosides)	11.9	624	‐	**607**, 589, 571, 541, 487, 316	253, 292, 337
**3**	Diosmetin 6‐*C*‐glucoside/diosmetin 8‐*C*‐glucoside	16.2	462	413, 371, **341**	**445**, 427, 409, 397, 367, 343	270, 343
**5**	Diosmetin 6‐*C*‐glucoside/diosmetin 8‐*C*‐glucoside	17.0	462	413, 371, **341**	**445**, 427, 409, 397, 367, 343	272, 338
	*Flavanone O‐glycosides*					
**4**	Naringin (naringenin 7‐*O*‐neohesperidoside)/narirutin (naringenin 7‐*O*‐rutinoside)	16.5	580	459, 313, **271**	447, **304**	283, 330
**8**	Poncirin (isosakuranetin 7‐*O*‐neohesperidoside)/neoponcirin (isosakuranetin 7‐*O*‐rutinoside)	22.2	592	575, **285**	449	283, 328
	*Flavone O‐glycosides*					
**6**	Neohesperidin (hesperetin 7‐*O*‐neohesperidoside)/hesperidin (hesperetin 7‐*O*‐rutinoside)	17.7	610	463, **301**	447, **304**	283, 327
**7**	Neodiosmin (diosmetin 7‐*O*‐neohesperidoside)/diosmin (diosmetin 7‐*O*‐rutinoside)	18.9	608	461, **299**	**463**, 301	253, 267, 347
	*Polymethoxyflavonoids*					
**9**	Isosinensetin	28.4	372		358, 343, 329, 312	244, 271, 342
**10**	Sinensetin	29.9	372		358, 343, 329, 312	242, 270, 333
**11**	Tetramethyl‐*O*‐isoscutellarin/tetramethyl‐*O*‐scutellarin	30.2	342		328, 313, 299	241, 270, 342
**12**	Nobiletin	31.3	402		**388**, 373, 343, 312	249, 271, 334
**13**	3,5,6,7,8,3′,4′‐Eptamethoxyflavone	32.3	432		**418**, 403, 385	253, 270, 342
**14**	Tangeretin	33.1	372		**358**, 343, 312	233, 271, 323

^a^ Product ions are generated by fragmentation of the [M − H]^−^ parent ion and base ion peaks are shown in bold.

^b^ Product ions are generated by fragmentation of the [M + H]^+^ parent ion and base ion peaks are shown in bold.

The quantitative analysis (Table 4) revealed that compounds **9**–**11** and **13** were present in this juice only in traces, while **12** and **14** were more abundant, with **12** present in higher quantity than **14**. Among flavonoid glycosides compound **6**, identified as hesperidin or its isomer neohesperidin, was found to be the most abundant compound in agreement with the literature (Russo et al., 2011; Wang et al., 2017), followed by compounds **11**, **4**, **7**, and **8** (Table 4).

**TABLE 4:** Amount, expressed as mg/L ± SD of fresh fruit, of main flavonoids identified in *C. reticulata* juice

No.	Compound	mg/L ± standard deviation of fresh juice
**1**	Vicenin‐2	40.1 ± 1.6
**2**	Lucenin‐2 4′‐methyl ether	14.6 ± 1.1
**4**	Naringin/narirutin	63.6 ± 5.5
**5**	Diosmetin 6‐*C*‐glucoside/diosmetin 8‐*C*‐glucoside	9.8 ± 0.3
**6**	Hesperidin/neohesperidin	570.2 ± 45.1
**7**	Neodiosmin/diosmin	93.0 ± 6.7
**8**	Neoponcirin/poncirin	36.6 ± 3.6
**12**	Nobiletin	89.4 ± 2.7
**14**	Tangeretin	63.9 ± 2.4
	Total amount	981.2 ± 69.0

*Note*: Compound numbers correspond to those listed in Table 3.

## 4 CONCLUSION

MJ obtained from *C. reticulata* fruits, collected in the *Horti Simplicium* of Pisa Charterhouse, present a high amount of flavanone glycosides and a number of polymethoxyflavonoids. Moreover, this work demonstrates that MJ possesses a marked nutraceutical value, useful in the management of hyperglycemia, dyslipidemia, and obesity typical of metabolic syndrome.

Taken together these results confirm that hesperidin, the most abundant flavanone, and the numerous polymethoxyflavonoids, including nobiletin and tangeretin, may be active metabolites responsible for beneficial effects of MJ. These results are in agreement with the literature, indeed antiobese effects have been reported for several methoxhyflavonoids; moreover, hesperidin and its aglycone hesperetin are recognized as active constituents of several type of *Citrus* juices, including orange juice (Xiong et al., 2019). Finally, active constituents of MJ have been demonstrated, for the first time, to be able to stimulate the secretion of irisin and its downstream mediators, PGC‐1α, PPARγ and UCP‐1, responsible for adipose tissue browning.

## CONFLICT OF INTEREST

The authors declare no conflict of interest.

## Data Availability

The data that support the findings of this study are available from the corresponding author upon reasonable request.
